# Time at Home during the COVID-19 Pandemic: Findings from Focus Groups with Hispanic Parents

**DOI:** 10.3390/children9050634

**Published:** 2022-04-28

**Authors:** Amber J. Hammons, Ryan Robart, Guadalupe Gonzalez

**Affiliations:** Department of Child and Family Science, California State University, Fresno, CA 93740, USA; rrobart@csufresno.edu (R.R.); ggonzalez@hindshospice.org (G.G.)

**Keywords:** COVID-19 pandemic, family time, family strengths, Hispanic, familism, resilience

## Abstract

The COVID-19 pandemic upended family life, forcing many families to reorganize their daily routines. Hispanic families have been especially affected by the pandemic, experiencing cumulative stressors and increased risks of contracting the virus, hospitalization, and morbidity. To date, there is limited research examining home life within Hispanic families during the pandemic. Given the extended amount of time for which families have been isolated at home together, identifying factors that may enhance or detract from well-being within the home is important in advancing efforts to support at-risk families. In this qualitative study, 29 Hispanic parents (primarily mothers) living in California participated in one of eight focus groups conducted in Spanish. Parents described activities and behaviors during the first year of the COVID-19 pandemic. The following six themes were identified using reflexive thematic analysis: (1) parents focused on family time; (2) children adapted to the changes of the pandemic; (3) parents and children engaged in physical activity; (4) children mainly entertained themselves with screens; (5) COVID-19 media coverage was accessible in the home; and (6) parents worried about the virus, and its effects on the future. While findings include anxiety around the virus and its attendant effects, family strengths were also present throughout the discussions. Public policy should consider ways to leverage family strengths to preserve family relationships and routines during future public health crises.

## 1. Introduction

The COVID-19 pandemic disrupted family life, with many parents finding themselves spending inordinate amounts of time with their children at home and reorganizing their daily routines. For almost all families, this type of stressor coupled with the sudden onset of the pandemic was likely to increase stress levels, at least temporarily, as families attempted to establish a new equilibrium. While long-term effects are unknown, the scientific community has begun to document a range of outcomes associated with the pandemic. A study of 101 countries found increases in social isolation and loneliness in adults from June to November 2020 [1], and studies conducted in Canada, the United Kingdom, and the United States, reported changes in parental mental health in the beginning of the pandemic [2,3], as well as a year later [4]. According to a national report conducted in February 2021 in the United States, both mothers and fathers desired more emotional support than they received during the first year of the pandemic [4]. American parents experienced more stress during the first couple of months of the pandemic [5], and adults encountered sleep problems, reported higher rates of drinking, and gained unwanted weight compared to prepandemic [4]. Studies using samples representing diverse countries around the world found evidence for weight gain in children too amidst a disruption of regular routines [6,7,8]. American children reported feeling bored, worried, anxious, and unhappy during quarantine [9], and some families experienced increases in family conflict [10,11,12] and changes in family functioning [13] in the early pandemic. While much of the research has focused on deleterious health effects, some studies have also reported positive outcomes, including increases in family connectedness during the lockdown period (e.g., [14]).

Racially and ethnically diverse populations have been especially affected by the pandemic in the United States. According to the U.S. Census Bureau’s Household Pulse Survey conducted between August and December 2020, 29% of Latino households (with children) experienced multiple co-occurring economic and health-related hardships due to the pandemic [15], and a report in September 2020 found that nearly three-quarters of child deaths consisted of Hispanic or Black children [16]. Hispanics in the United States are overrepresented in essential jobs, magnifying their risk of contracting COVID-19 [17,18,19], and negatively influencing various aspects of home life. One study found that Latina mothers’ stress was related to worries about contracting the virus and economic cutbacks in the early pandemic [20]. Hispanic parents reported higher levels of financial stress in the early pandemic than White and non-Hispanic Black parents, which included major life stressors such as losing a job [18]. Hispanic parents also experienced more difficulty finding childcare than their Caucasian counterparts in both the early part of the pandemic [18], as well as a year later [21] when many schools began to reopen across the United States, which can contribute to elevated levels of family discord. A prepandemic survey highlighted the centrality of family in Hispanic women, finding that the leading cause of stress is concern about other family members’ health [22]. The pandemic intensified this anxiety, as parents were likely to worry about their families contracting the virus, and most parents were concerned about their child’s well-being [9] and academic performance [23] during this time. Parental worry and stress can spillover into the parent–child relationship, affecting overall family functioning, and one study conducted in the United States found that parental distress predicted child behavior problems early in the pandemic [24].

According to family systems theory, the family is viewed as an entity made up of multiple subsystems that are interdependent and interconnected [25]. If there are problems in one subsystem (individual or dyadic), there is potential for the problems to spill over into another subsystem. The pandemic created conditions of high stress for many families, including economic instability, changes in working conditions, reduced social support, and disruption of normal family routines, with risks to families potentially exacerbated by prior circumstances such as mental health or marital problems. Cascade effects may occur if parents become overwhelmed in the face of cumulative stressors generated by the pandemic, potentially resulting in poor functioning that then spills over into parenting, weakening the parent–child relationship, and over time negatively affecting child well-being [26]. The unprecedented COVID-19 pandemic transformed what initially appeared to be an acute stressor into a chronic one, as the virus became characterized by its long-term persistence and lack of predictability, magnifying the risk to family well-being. Chronic stressors call on the family as a functional unit to use family processes to adapt (or maladapt) [27], but family processes are particularly vulnerable during a pandemic, as regular routines and rituals that structure family life are upended. While the pandemic puts families at risk of negative outcomes, strengths also exist within families that may be harnessed during times of stress. Reliance on modified family routines and attention to nurturing close relationships can solidify a sense of familial belonging and connectedness during times of uncertainty, ultimately promoting positive adaptation [26].

Familism, a cultural value that places a strong emphasis on the importance of family relationships and interconnectedness, is especially important within Hispanic culture [28], and may offer protection during times of stress, providing families with a buffer against some of the effects of long-term stressors. Indeed, familism has been found to have both general and stress buffering effects on well-being and health, with familism predicting lower levels of loneliness and depression, and under conditions of high stress, high familism has been associated with self-esteem and subjective health [29]. In a qualitative study conducted in May 2020 in the United States, 10–14 year-old Hispanic youth expressed gratitude for their families and shared that relationships with their mothers and siblings became closer during the first few months of the pandemic [30]. Another study found that better family functioning during the stay-at-home order was related to fewer mental health symptoms for Latino youth in the United States who experienced mental health problems prior to the pandemic [31]. Close and supportive relationships are at the core of familism, and perceived social support is the mechanism through which familism has been hypothesized to contribute to psychological health [32]. Feelings of familial support may be disrupted during the COVID-19 pandemic as families deal with a multitude of stressors, and when stressors pile-up and become overwhelming, the benefits of familism may be limited [32].

The pandemic introduced many avenues of potential stress and hardship for families. In addition, economic challenges, cyclical lockdowns, remote schooling, and frequently changing mandates have compounded the complexity of balancing family life. In the context of the COVID-19 pandemic, within which families have been confined together at home for extended periods of time, identifying potential risk and protective factors within the home is critical in advancing efforts to support families. There is limited research looking at how Hispanic families spent their time at home during the first year of the COVID-19 pandemic. Given the disproportionate effects on Hispanic families in the United States, understanding how families spent their time, how they engaged with one another, and how they adjusted at home during the pandemic can be helpful in guiding public health messaging, directing resources, and informing health promotion interventions. The objective of this exploratory qualitative study was to address this research gap by describing activities and behaviors families were engaged in while home, and how families were adjusting during the first year of the pandemic.

## 2. Materials and Methods

Parents (primarily mothers) from California participated in 1 of 8 virtual focus groups conducted in Spanish during December 2020–February 2021. Most parents were residing in the Central Valley at the time of the study. Focus groups were conducted virtually via Zoom due to state lockdowns and university regulations preventing in-person meetings. Parents had to have a child between the ages of 5 and 18, and access to the internet, to be eligible to participate in the study. Families were mainly recruited from a prepandemic contact list of parents interested in learning more about Hispanic family-related health programs, who had been approached at grocery stores, flea markets, and churches frequently used by Hispanic families in urban areas. Additional recruitment strategies included word-of-mouth, social media, an email sent to students in the Child and Family Science program at the university, and snowballing. Parents received a $25 gift card for their participation in the study.

This portion of the study was part of a larger focus group study on the impact of COVID-19 on families. The larger study was divided up into three parts and included a focus on the family food environment [33], screen time [34], and family life. This paper focuses on part III, family life, which only includes focus groups conducted in Spanish. The study was approved by the California State University, Fresno’s Child and Family Science’s Department Review Committee (protocol #0002, 11/24/20). A semi-structured interview guide was created based on previous research on Hispanic families exploring family time and challenges, and incorporation of the literature on family functioning, process, and crisis (e.g., [35,36,37]). For this portion of the study, ten questions inquired about parenting changes, family time, routines, behavior changes within the family, parent and child health, remote/virtual school, and pandemic concerns. Sample questions included, “How has your family been spending time during the pandemic?”, “Overall, how has your child responded to the pandemic?”, and “Has your parenting changed during the pandemic? If so, how?” Parents also completed a short (approximately 5 min) anonymous parent self-report survey that asked about demographic information including place of birth, age, number of children, generational status, place of residence, health status, language spoken, and vaccine hesitancy.

Focus groups were conducted by a trained member (G.G.) of the research team with prior experience conducting focus groups and working with Hispanic families in the local community. The focus groups lasted an average of 57 min and an average of 3 parents participated in each group. They were audio-recorded and recordings were transcribed verbatim into the original language, and then translated into English by three proficient Spanish/English bilingual research assistants (A.G.L., N.G., E.P.). English translations were back-translated into Spanish to compare to the original transcripts. When discrepancies were present, they were noted, discussed, and resolved by consensus. Back-translations were triple-checked for accuracy.

Reflexive thematic analysis [38], grounded within a phenomenological framework, was used to analyze the transcripts. Two researchers (A.J.H. and R.R.) using an inductive approach, began by immersing themselves in the transcripts, reading and studying them. Researchers took notes documenting their thought processes, insights, and reflections on potential codes in reflexive journals. Once researchers were fully familiar with the transcripts, codes were created and applied, first independently; then jointly. Codes were refined throughout the process, and when new ones were identified, were reapplied to transcripts. Researchers discussed the coding process as coding took place to clear up any discrepancies and to explore the transcripts in more depth. When discrepancies arose, researchers discussed and resolved them by consensus. Additionally, the lead researcher checked in with the project coordinator (G.G., who conducted the focus groups) and research assistants for discussion, reflection on notes, and consideration of possible saturation points to help ensure integrity of the data. Once transcripts were coded completely, themes were identified based on patterns of meaning. Member checking was also used to ensure integrity of the themes and interpretations. As a final check, researchers went back to the data to confirm that study decisions were grounded in the data [39]. Study researchers then selected representative quotes. Participant quotes are denoted by a focus group number and then followed by an arbitrary number assigned to each participant in the findings below. Data were organized via qualitative analysis software, Dedoose Version 9.0.17 (Sociocultural Research Consultants LLC.: Los Angeles, CA, USA).

## 3. Results

Twenty-nine Hispanic parents (*n* = 28 mothers) participated in the study. All parents lived in California, most in the Central Valley (97%). The average age of the parent was 37.00 (SD = 8.13), and the average age of children was 11.23 (SD = 5.83). Children were attending school remotely when the study was conducted. Most (90%) of the parents were born in Mexico and 59% were first-generation immigrants. Slightly less than a quarter (21%) of the participating parents were employed, and of those, two-thirds were working remotely. The majority of the sample made less than USD 50,000 a year. Almost half (48%) lived in a household with an essential worker, or were themselves one. See Table 1 for medians and interquartile ranges (IQR), and additional participant characteristics.

Six themes were identified: (1) “Reinforce a little more family time”—Parents focused on family time; (2) “They grew used to it”—Children adapted to the changes of the pandemic; (3) “We go for a walk”—Parents and children engaged in physical activity; (4) “Phone, and computer”—Children mainly entertained themselves with screens; (5) “This is real”—COVID-19 media coverage was accessible in the home; and (6) “I fear that everything is not going to be the same”—Parents worried about the virus, and its effects on the future.

**Theme 1: “Reinforce a little more family time”: Parents focused on family time.**

The majority of parents shared that they had made positive changes during the pandemic, with most saying that this meant spending more time together as a family. This manifested most frequently in watching movies and TV together, playing games, going on family walks, and sharing meals. Dinners were a time to connect with family members and socialize at the table. Families also found more ways to become involved in activities that included all family members, and family time often merged with exercise, with family members doing something active together outside, usually going on a family walk. Parents tended to describe the family time with fondness, “the truth is, something I thank the pandemic very much for is this family time because I spent a lot of time alone” (FG5.5), and, “although this situation has affected many aspects, I believe for me personally it has brought my family together more” (FG8.1). For others, it also meant feeling less rushed, being able to pay more attention, and show greater responsiveness, as this mother shared:

*“I used to be in a rush, would leave to go to work, I would just say I love you in a rush, good morning in a rush. However, not now, with all the patience, “good morning, I love you”, and I give a lot of hugs, I tell him how much I love him, I show him all the love I have for him, and before I was in a rush, and not anymore. That changed a lot.”*

(FG7.4)

**Theme 2: “They grew used to it”: Children adapted to the changes of the pandemic.**

The majority of parents shared that overall their children were doing okay and had adapted to the changes of the pandemic. At first, there was confusion around the unknown but with time children adjusted to the new routines. This mother highlighted the ease of the transition for her children, “My kids are troopers too, and I think they kind of adjusted pretty well to the pandemic, and I am actually surprised by that” (FG4.3). Parents talked about positive aspects of the adjustment, such as enjoying being at home and being together as a family. Eventually, children came to understand the reasoning behind why they could not do the same things they did prepandemic and had come to accept the situation, as this mother described:

*“They responded, at first negatively, at first, they would get upset and say, “ayy not this pandemic” and complain, they had been telling me that they want to go to school, that they did not like to be here, but now they say it is for our own good to be [home]…it’s for our own good to be like this online.”*

(FG5.3)

Since all of the children had been attending school remotely for the previous 9–11 months (depending on when parents participated in the study) and spent most of the day attending school on their computers, parents focused on how their children adapted specifically to the transition to remote schooling. Similar to children’s overall adjustment to the pandemic, parents discussed how children found the move to be challenging at first, that it was quite difficult at times, with children struggling, but that with time children got into a routine and found that it became easier. Some parents shared that they started to see improvements in behavior and grades after an initial adjustment period. This mother described how it was an adjustment even for her, that the situation was made more challenging due to her lack of understanding of the virtual school process, but that it got better:

*“Well, my children have done well. At the beginning, my girl entered first grade and she did not know much about technology. So, if we have work, and it is difficult for me because I do not know how to send the homework, then I have to wait so that my child has time to be able to send homework and open the applications she needs to do. So, mine has gone well, the problem has been how to deliver the homework of the youngest girl, it has been complicated for me until now.”*

(FG8.2)

A few parents expressed concern that their children had adjusted perhaps too well, as this mother shared, “However, now he is accustomed and I tell him that school is going to start and he says no, he is comfortable at home. He is good.” (FG7.4). Several of the parents mentioned that their children were doing fine now, but that they often mentioned missing their friends and were ready for the pandemic to be over. In contrast, while the majority shared that their children had adapted to the situation, several parents added that on the whole their children were not faring well and that they had observed negative changes in their children as a result of the pandemic.

**Theme 3: “We go for a walk”: Parents and children engaged in physical activity.**

The majority of parents considered themselves to be active during the pandemic. The most common parental exercise was walking, but several parents shared that they preferred exercise videos on YouTube. Some parents discussed how cleaning the house was the exercise that they regularly engaged in, “the chores around the house, for me, that is exercise” (FG2.2).

For most children, isolating at home changed the type, intensity, and frequency of physical activity they engaged in. Parents shared that children were active during the pandemic when they would exercise or dance indoors, sometimes using a physical activity video game or video online, and when they would go outside, which included unstructured activities and playing, bike riding, games, and walking with the family. Walking was an activity families tended to do together most frequently, around the neighborhood or in the park. Some parents talked about how their children would exercise during virtual school, which varied by school and grade level, but many parents were not sure what types of virtual physical activities children were actually doing. While most parents felt their children were getting some exercise, parents perceived that overall physical activity levels for children were lower than prepandemic, and some felt that they were much lower. This parent commented:

*“It [physical activity] would be very little. Just on the days of the week that they do virtual PE, and on the weekends when they can get together with their cousins. From time to time they have video games that include physical activity, but usually nothing compared to what they did before in school, or when we could go out a little bit more frequently to the park. So, it has decreased a lot.”*

(FG8.3)

**Theme 4: “Phone, and computer”: Children mainly entertained themselves with screens.**

Parents shared that their children mostly entertained themselves by spending time on their cell phones and computers during the pandemic. They talked about children spending time watching TV and movies, making videos on Tik Tok, and using game consoles such as Nintendo or Xbox. One mother described it as “an increase in the interaction between themselves [and screens] as they do not have other children with whom they are playing or talking, so this is basically the main thing [for entertainment]” (FG8.3). Another parent shared his experience with his children’s screen time: “So, the adaptation has been forced, not by choice. The only thing is the electronic thing that is pulling them, it is the only thing they like here…” (FG8.1). Other frequently mentioned entertainment activities included playing outside, spending time with family, and drawing.

**Theme 5: “This is real”: COVID-19 media coverage was accessible in the home.**

The majority of parents did not restrict COVID-19 news in their households, it was open for discussion. Some parents talked about how the news was helpful in the beginning of the pandemic because, “I did not know how to explain well what it was” (FG8.2), and it was useful to know the symptoms. It also helped some parents and their children to realize the seriousness of the situation, “At the beginning of the pandemic, we were not very convinced that this was real but then to look at so many cases and to come to us ourselves was that… we take caution, and then this is real” (FG5.3). Several families shared that their children were not very interested in the news, or that they became bored with hearing about the pandemic all the time. Still, a couple of parents remarked that they began to restrict access because it was overwhelming and too negative. Sometimes this was for themselves, but usually it was a decision to protect the whole family. One mother commented:

*“Additionally, sincerely since this started, not only the children but we also got super nervous. During that time the news would show the empty shelves, they looked at each other when they emptied the shops and my daughters would say to me, “Mommy, and what are we going to do without food, mommy what are we going to do without this?”. It was a concern for them and for us, and more them because they understand to a certain extent, they get to a point that they understand, their mind shoots further than their knowledge and you do not know to what extent it can affect them or to what extent they cannot. Additionally, they get carried away by everything that is in the news, or what they said practically in the news and they imagined themselves as their worst, and one did not, one said, “okay, because if we cannot get things in this store, we can get it in this one”. Or we find it easier to look for the things that were said in the news that were gone, and for them they get affected to a certain point, so after that we would not let them watch the news.”*

(FG4.2)

**Theme 6: “I fear that everything is not going to be the same”: Parents worried about the virus, and its effects on the future.**

Parents talked about how the virus had changed life so much that a return to the way things used to be may not happen. Parents expressed concerns about their children returning to school and what that might look like. Some parents feared that children may have to wear masks indefinitely, that they may struggle with new rules around the virus at school, and that schools might mandate COVID-19 vaccinations for children. Parents also discussed the persistent nature of the pandemic and how it felt never-ending at times. One mother shared,

*“I keep thinking, what worries us the most is that this is going to go on for many years, so that my girls are going to have to get used to it, they are going to have to grow up in a world like this, with fear, always fear in the back of our minds where they will not be able to join peer parties, meet their peers, their teachers, who will always have to be on the computer and more that the virus would come back. Thank God no one has passed away, no one immediately from our family, but if something were to happen due to this pandemic, I think it would be very difficult to talk to my girl, or that we get sick again, I think it’s the fear more of this, it’s going to last and lose years from her childhood.”*

(FG4.1)

They also feared getting the virus, “I think about what would happen if I got it, it gives me terror” (FG1.2). Some were regularly vigilant, and worried about how their families would respond if infected. This mother expressed her concerns, “Yes, what worries me is that the virus will always be here since it started, and when will we get it, because we will get it sooner or later, and what worries me is when and how hard of a sickness will we get.” (FG7.1).

Parents were asked in an anonymous survey about whether they intended on getting their families vaccinated when a COVID-19 vaccine became available. As a way to further contextualize the information presented here, the results from the survey are included. About one-third (34%) of the parents said they planned to get their families vaccinated when a vaccine became available, 10% said they did not plan to have their families vaccinated, 52% of parents said they did not know if they would have their families vaccinated, and one person declined to respond.

## 4. Discussion

The objective of this study was to learn how Hispanic families were spending their time at home and adjusting during the first year of the COVID-19 pandemic. Many of the parents shared that they focused more on family time and the majority perceived that their children were doing okay and had adapted to the changes of the pandemic. A focus on nurturing relationships, adapting to challenges, and establishing/maintaining routines were present in the discussions, and these elements characterize strong families [40]. A characteristic of family resilience includes the ability of families to maintain routines and rituals flexibly [41]. Families discussed how the transition to remote schooling became easier once children adjusted to the new home routine, and research has found that establishing predictable family routines can help foster a positive emotional climate [42], which may have been particularly important during this time. Lockdowns may have created opportunities for families to find novel ways to be together, and parents in the current study shared that the increased time spent together was a pleasant aspect of the pandemic. This is consistent with other studies that found that families experienced more emotional closeness and enjoyment of family time during the pandemic [2,14,43]. These patterns of positive interactions may spill over into other areas of family life, strengthening the family unit [27]. A multi-country study found that the number one lifestyle change that parents hoped to see continue post-pandemic was more quality family time [44]. One future direction of research may be to follow-up with families to see how successful they are at accomplishing this goal, and for public health efforts to find ways to help facilitate this, as family connectedness may help to buffer families from other areas of stress [45].

While some studies reported increases in positive family experiences and closeness during the early parts of the pandemic (e.g., [46,47]), others reported increases in family conflict (e.g., [10,11,12]) and struggles with maintaining family routines (e.g., [48]). The severity of the stressors as well as the cumulative effect seem to be critical in understanding their role in increasing conflict [10,26], but also examining the seemingly paradoxical nature of additional family time being simultaneously pleasant and conflictual for some families (e.g., [2]) may yield interesting insights into family life during periods of stress and uncertainty. Qualitative research may be a useful tool in helping to unpack these nuances. When parents in the present study discussed the increase in family togetherness, which often centered around family routines, the positive aspects of the additional time tended to be emphasized. Quantitative research may be helpful in examining if families high in feelings of connectedness are different from families lower in connectedness and determining the extent to which these relationships may be protective in the context of the pandemic. Familism may serve as a buffer, helping families to feel supported during the pandemic and mitigating the impact of other stressors, but more research is needed to explore this relationship further.

While the pandemic forced restrictions on physical activity opportunities for many, parents in the present study shared that they still found ways to be active. The COVID-19 pandemic challenged families to flexibly adapt and develop new routines, which parents in this study shared that they did by finding things to do as a family to be active, which usually meant going on walks together. In prepandemic research on Hispanic families and physical activity, some parents shared that they prefer to exercise alone [49], but circumstances during the pandemic may have encouraged families to exercise together and altered family dynamics, as roles and resources may shift in the face of stressful events [41]. However, it was unclear in this study if families who engaged in family exercise together increased their overall family physical activity relative to prepandemic, and if exercise helped reduce stress levels; this needs to be explored further. This information could be important for culturally tailored health-promotion programs because programs can build on the momentum of what worked for families during this time, which may help to sustain and enhance these joint healthy behaviors going forward. Virtual programming that encourages families to be active together and includes age-appropriate family games that can be played indoors and outdoors may be beneficial and welcomed by families. Notably, when families shared changes they made since the pandemic began, healthy eating was seldom mentioned. Diet is a crucial player in overall health and weight status, and public health messaging and health-promotion programs should focus on promoting healthy eating during potential lockdown periods, especially given the high rates of obesity in the Hispanic population and the concomitant effects related to COVID-19 [50,51,52].

Screen time use has also been linked to higher obesity rates in Hispanic populations [53] and Hispanic adolescents spent more time on screens than White and Asian adolescents in the United States during the first year of the pandemic [54]. Much of children’s screen time was devoted to remote schooling, but parents reported in this study that children mainly spent their recreational time on screens as well, which also captured some family time, as parents shared that family time consisted of watching TV and movies together more often. While screen time is hypothesized to present a risk in part because it can displace healthier activities (e.g., [55,56]), future research may include exploring the perceived benefits associated with joint/family screen time activities, such as bonding and feeling connected to one another, and the ways in which family screen time evolves post-pandemic. Understanding Hispanic parental views of the benefits of family screen time post-pandemic may help to shape how culturally tailored health-promotion programs address screen time and obesity prevention in Hispanic populations.

Family connectedness is highly valued in Hispanic culture, and it is a strength that families may rely upon when dealing with chronic stressors [57]. A national prepandemic survey revealed that Hispanics use more healthy coping skills and engage in fewer unhealthy behaviors to manage stress compared to the general U.S. population [22]. Healthy behaviors included relying on family members for support and exercise, both of which were represented in the present study. A qualitative study on Hispanic youth in the United States during the early pandemic found similar findings with regard to coping, with adolescents reporting exercising and turning to their families for support [30]. Parents in the present study shared that they worried about their children during the pandemic, and about their child’s future in the face of unknowns. They expressed anxiety about how the situation would unfold in schools and how to cope in a new and ever-changing world, but they were also open about the pandemic situation, unrestricting news and discussions within the household. If the news became too distressing, parents discussed limiting access or avoiding it altogether as a way to cope as a family. However, it is unknown in this study whether pandemic-related anxieties generated significant levels of distress and whether this was indeed buffered by family strengths. More research is needed to understand the degree to which family strengths may be protective against areas of family stress, including financial strain in families, induced by the COVID-19 pandemic.

This study included several limitations. The impact of the pandemic on families’ economic situation was not explicitly explored in the focus groups, which is likely to influence parental stress and family functioning, potentially limiting the benefits of close relationships. Directly inquiring about the role of economic impacts may reveal a more comprehensive view of the interplay between family interactions and financial challenges. Additionally, fathers’ perspectives were largely lacking in the present study as only one father participated. A nationally representative survey conducted in the United Kingdom found that fathers spent more time with their children and reported closer relationships with them during the lockdown period [58]. Hearing from more Hispanic fathers about how they experienced time at home during the pandemic could help to strengthen the findings and more accurately inform recommendations. A prepandemic qualitative study found that Latino fathers prefer activities that combine family togetherness and physical activity to screen-time activities [59]. Child screen-time activities have been at an all-time high during the pandemic, and understanding how Hispanic fathers navigated these areas within the home could further inform health-promotion programming. The current sample was also a relatively healthy one, with 89% of parents self-reporting being in good health; a less healthy sample is likely to experience the pandemic differently. Lastly, we chose to examine families broadly, with all family members included, which prevented us from examining how the pandemic affected specific developmental periods. Children of varying ages have been affected differentially by the pandemic (e.g., [60,61,62]), and these differences could not be fleshed out in the present study.

This study adds to the current literature a focus on Hispanic family home life, and sheds light on how behaviors and activities at home may help inform public health efforts to support families, but it is only a first step. As research on the COVID-19 pandemic continues, future directions include examining whether family connectedness and routines buffer against COVID-19 related stressors, and examining whether and how family strengths may predict other healthy outcomes. Building on the momentum of the desire to maintain closeness and to incorporate physical activity in family behaviors may help to preserve important family routines and enhance family functioning. Public policy should consider a focus on promoting healthy behaviors and directing resources to families to facilitate healthy relationships and routines during future health crises.

## Figures and Tables

**Table 1:** Participant Characteristics.

Median Parent Age (IQR)	38.00 (31.00–43.00)
Median Child Age (IQR)	12.00 (7.00–16.00)
Median Number of Children per Family (IQR)	3.00 (2.00–4.00)
Married or Cohabitating, *n* (%)	23 (79)
Parent Gender, *n* (%)	
Mother participants	28 (97)
Father participants	1 (3)
Resided in Central Valley, California, *n* (%)	28 (97)
Parent Language Spoken, *n* (%)	
Speaks only Spanish	10 (35)
Spanish better than English	8 (28)
Spanish and English equally well	8 (28)
English better than Spanish	3 (10)
Parent Education, *n* (%)	
Less than high school	14 (48)
High school	6 (21)
Technical school	4 (14)
Bachelor’s degree	2 (7)
Master’s degree	2 (7)
Declined to respond	1 (3)
Parent Health Status, *n* (%)	
Excellent	3 (10)
Very good	2 (7)
Good	21 (72)
Fair	3 (10)

## Data Availability

Data are confidential to protect the privacy of research participants.
